# Work hours, weekend working, nonstandard work schedules and sleep quantity and quality: findings from the UK household longitudinal study

**DOI:** 10.1186/s12889-024-17762-0

**Published:** 2024-01-27

**Authors:** Gillian Weston, Afshin Zilanawala, Elizabeth Webb, Livia Carvalho, Anne McMunn

**Affiliations:** 1 ESRC International Centre for Lifecourse Studies, Research Department of Epidemiology and Public Health, University College London, 1-19 Torrington Place, London, WC1E 6BT UK; 2 Department of Social Statistics and Demography, University of Southampton, University Road, Southampton, SO17 1BJ UK; 3 Age UK, 7th Floor, One America Square, 17 Crosswall, London, EC3N 2LB UK; 4 Department of Clinical Pharmacology, William Harvey Research Institute, Queen Mary University of London, Charterhouse Square, London, EC1M 6BQ UK

**Keywords:** Sleep disturbance, Sleep duration, Work hours, Long hours, Part-time hours, Nonstandard work schedules, Weekend work, Atypical temporal work patterns, UK household longitudinal study, Understanding society

## Abstract

**Background:**

Atypical temporal work patterns such as working longer than the standard 35–40 h/ week, weekend working, and nonstandard work schedules (i.e. outside of the typical 9–5, including but not restricted to shiftwork) are increasingly prevalent in the UK. Aside from occupation-specific studies, little is known about the effects of these atypical temporal work patterns on sleep among workers in the UK, even though poor sleep has been linked to adverse health problems, lower workplace productivity, and economic costs.

**Method:**

We used regression models to investigate associations between three types of atypical temporal work patterns (long and short weekly work hours, weekend working, and nonstandard schedules) and sleep duration and disturbance using data from over 25,000 employed men and women from 2012–2014 and/or 2015–2017 in the UK Household Longitudinal Study, adjusting for potential confounders and psychosocial work factors.

**Results:**

We found that relative to a standard 35–40 h/week, working 55 h/week or more was related to short sleep (less than 7 h/night) and sleep disturbance. Working most/all weekends compared to non-weekends was associated with short sleep, long sleep (more than 8 h/night), and sleep disturbance, as was working nonstandard schedules relative to standard schedules (fixed day-time schedules). Further analyses suggested some gender differences.

**Conclusions:**

These results should prompt employers and policymakers to recognise the need for rest and recovery, consider how the timing and scheduling of work might be improved to better support workers’ health and productivity, and consider appropriate compensation for anyone required to work atypical temporal work patterns.

**Supplementary Information:**

The online version contains supplementary material available at 10.1186/s12889-024-17762-0.

## Background

### Sleep

Although there are inter-individual differences in the amount of sleep needed and in subjective assessments of sleep quality [1], adults are recommended to sleep at least seven hours/night [2], fall asleep within approx. 30 min, wake for no more than five minutes once per night, and feel satisfied with their sleep [3]. Studies show a u-shaped association between sleep duration and poor health [2], and evidence that insufficient sleep (habitual short sleep duration) and sleep problems (e.g. difficulties initiating or maintaining sleep) are associated with mental and cognitive health problems, and chronic diseases [4]. Although there is more evidence for the links between short sleep and health [2], a systematic review and meta-analysis recently concluded that long sleep durations (> 9 h/night) are significantly associated with mortality and poor health outcomes including coronary heart disease and cardiovascular disease [5]. Poor sleep is also linked to work-related injuries [6], and productivity losses estimated in excess of £40bn/annum in the UK [7].

Yet poor sleep is common [8], particularly on workdays compared to weekends [9] because workday sleep is often curtailed by alarm calls due to work scheduling [10]. Sleep deprivation can leave workers vulnerable to smoking, alcohol consumption, and depressive symptoms, which can further hinder sleep [10].

### Atypical temporal work patterns

Like sleep, atypical temporal work patterns are associated with poor health [11–13]. Atypical temporal work patterns are timings and intensities that deviate from the typical 9–5 full-time, Monday to Friday pattern, e.g. part-time, long hours and nonstandard work. Nonstandard work comprises of nonstandard schedules (e.g. early mornings, evenings, nights, and rotating shifts) and nonstandard days (i.e. weekend working) [14]. Some researchers differentiate between the two because weekends are usually deemed rest days, and some differentiate between their frequency because infrequent weekend working is often a symptom of overtime working, whereas frequent weekend working is associated with lower-skilled occupations [15].

These patterns are characteristic of the modern economy - due to 24/7 operating systems and fluctuating demands for goods and services [16]. Sometimes they provide a pay premium and help workers reconcile work and family demands [17]. In the UK, approximately 25% of men and 10% of women work 45 + h/week, and 12% of men and 40% of women work fewer than 30 h/week [18], 37% work evenings, 16% at night, 20% have shiftwork, and 31% work weekends [19]. Data on how many workers combine these patterns is scarce, but we note that healthcare workers combine shiftwork with long hours [11], and 30% of Swedish workers combine part-time hours with nonstandard schedules [20].

### Atypical temporal work patterns and sleep

Working when the biological drive to sleep is strongest (such as night-times and early mornings) can disrupt the circadian rhythm [21]. Stress relating to psychosocial work factors, such as time pressures, job demands and work-life imbalance, can contribute to poor sleep [22], whereas work demands may prompt individuals to forgo some sleep time to get the work done [23]. Indeed, time-use studies found long and irregular schedules were associated with sleep reductions, and every hour increase in working time was associated with up to 14-min less sleep [23, 24]. Nonetheless, these studies may have overestimated sleep duration because they tended to assess time spent in bed rather than time sleeping.

Many researchers rely on self-report sleep data from psychometrically-validated questionnaires, such as the Pittsburgh Sleep Quality Index (PSQI) [25], because they are practical for use in large samples, are good proxies for actigraphy (activity monitors), and do not necessitate the complex requirements of polysomnography (collection of physiologic data during sleep) [26]. Several cross-sectional and prospective studies have used such questionnaires, reviews of which concluded that atypical temporal work patterns were associated with sleep disturbance and insufficient sleep [27–31]. However, this research tended to focus only on specific patterns, such as nightshifts, and omitted weekend working. Usually, it did not account for overlapping atypical temporal work patterns even though combinations such as long and irregular hours can be an obstacle to good health behaviours and wellbeing [32]. Although the number of working hours may be a predictor of sleep duration [33], and several studies investigated long working hours, the operationalization of long hours differs across the studies making comparisons difficult. Furthermore, despite the high prevalence of part-time working among women, there is scant research on the effects of part-time hours on sleep.

Moreover, many studies have omitted women. Yet, men’s and women’s experiences of work can differ because they receive different entitlements, and their duties, responsibilities and working styles often vary [34]. Also, women are less likely than men to work overtime, but more likely to have flexible work arrangements (such as reduced hours, term-time working and job-sharing), and to combine paid work with unpaid domestic activities [35]. Furthermore, there are gender differences in sleep. Women tend to sleep longer than men, but experience more problematic sleep [36]. There are also gender differences in job types, with women less likely than men to have high quality jobs and high-paying professional and managerial occupations [35]. And although sleep quality and duration varies among occupational populations [37], study samples often comprised of white-collar workers and/or specific occupational settings (e.g. the public sector, healthcare, transport, and manufacturing).

Additionally, the literature mostly focuses on two regions – East Asia and Scandinavia. Studies from East Asia usually found associations with poor sleep, whereas the findings from Scandinavia were mixed. But sleep is influenced by country and cultural context [38, 39]. In East Asia, there is a risk of death from overwork and there is a common view that sleep can be sacrificed to achieve success [27]. In Scandinavia, sleep is longer, working hours are shorter, and welfare state provision and employment protections are higher. Contextually, the UK differs to both regions on labour market norms, welfare state provision, and legislative protection [40, 41]. Here relatively little is known about sleep in relation to these work patterns, particularly for the general workforce. Studies on atypical temporal work patterns and long sleep are particularly rare.

### The current study

To address these shortcomings, we aimed to investigate the associations between atypical temporal work patterns and sleep using data from a large, nationally-representative sample of the UK population, to include men and women, to adjust for a range of covariates including health, and to account for overlap between work patterns. We assessed both sleep duration (including short and long sleep) and sleep disturbance (a multi-dimensional measure of sleep quality) with three types of atypical temporal work pattern: (1) weekly work hours which are longer or shorter than the standard 35–40 h/week; (2) nonstandard work schedules which deviate from standard (9–5 type full-days or half-days) schedules; and (3) weekend working relative to non-weekend working. We also asked, (4) does the strength of the associations between the work patterns and sleep change when we account for overlapping work patterns? (5) are there gender differences in the associations between the work patterns and sleep?

## Methods

### Data and sample

The UK Household Longitudinal Study (UKHLS), ‘Understanding Society’, follows people diverse in age and employment status, living in around 40,000 UK households. Data are publicly available [42], with collection approved by the University of Essex ethics committee [43]. At wave four (2012–2014) data was gathered on sleep, weekly work hours, weekend working, and nonstandard schedules, and at wave seven (2015–2017) again on sleep and weekly work hours (but not the other two work patterns). We created two samples. The first pooled the wave four and seven data (‘pooled sample’) to investigate weekly work hours only. The second was restricted to wave four data (‘w4-only sample’) to investigate weekend working and nonstandard work schedules separately and jointly with weekly work hours. Samples were limited to respondents who were working in employment or self-employment (thus excluding economically inactive individuals including full-time students and retirees), because we were interested in assessing how features of work influence sleep. This gave us 48,990 observations from 28,137 participants for the pooled sample, and 25,605 participants for the w4-only sample.

These samples had similar amounts of missing data, with item missingness ranging from 1.4%-10.1% on our exposure variables, 8.4%-9.3% on our outcome variables, and < 0.01–28.0% on our covariates (health behaviour variables had the largest amount of missingness) (shown in Additional file 1). To reduce potential bias and improve efficiency in parameter estimation, we applied multiple imputation by chained equations (MICE) and imputed 46 datasets for each analytic sample. MICE assumes that data is missing at random, which is common in epidemiological studies [44]. The imputation model included all analysis and auxiliary variables. Complete case results (available on request) were substantially in line with imputed results.

### Measures

#### Sleep

At waves four and seven, data were gathered on sleep using questions from the PSQI. For our measure on sleep duration, we coded the number of hours participants usually slept per night (per day for nightshift workers) into categories: < 7 h ‘short sleep’, 7–8 h ‘regular sleep’ (reference category), and ≥ 9 h ‘long sleep’. Our categorisation is consistent with previous work-related epidemiological studies [45], the recommended sleep duration for adult optimal health, and evidence of non-linear associations between sleep length and health outcomes [24]. Our measure of sleep disturbance comprised of three items: sleep latency (trouble getting to sleep within 30 min); sleep maintenance (waking in the middle of the night or early morning); and sleep quality (respondent’s subjective rating of their sleep quality overall). The first two were scored from 1 ‘not during the past month’ to 5 ‘more than once most nights’, whereas sleep quality was scored 1 ‘very good’ to 4 ‘very bad’. The three scores were standardised into z-scores, then combined and grouped into quartiles - the upper quartile represented sleep disturbance [46]. This aligns with the American Psychiatric Association’s DSM-V diagnosis of insomnia disorder relating to sleep problems [47].

#### Atypical temporal work patterns

We had three measures of atypical temporal work patterns. Our first measure, weekly work hours, summed the number of hours participants worked on average per week, overtime hours in a normal week, and hours from any second jobs, then divided total hours into four categories: < 35 h (part-time), 35–40 h (full-time—reference category), 41–54 h (long hours), and ≥ 55 h (extra-long hours). This categorisation was consistent with the World Health Organization’s research [48]. The second measure, a binary variable for nonstandard schedules, categorised what time of day participants worked. Standard schedules (reference category) represented responses ‘during the day’, ‘mornings only’, ‘afternoons only’. Nonstandard schedules represented ‘evenings only’, ‘at night’, ‘both lunchtimes and evenings’, ‘other times of day’, ‘rotating shifts’, ‘varies/no usual pattern’, ‘daytime and evenings’ and ‘other’. Our third measure, weekend working, retained the three response options to the question ‘do you ever work weekends?’: ‘no weekend working’ (reference category), ‘some weekends’, and ‘most/all weekends’. To account for overlapping work patterns we mutually adjusted for the aforementioned three types of work pattern. In a supplementary analysis our three exposure variables were combined then divided into 24 categories—one comprising of no atypical patterns (full-time with standard schedules and non-weekends—reference category), six containing a single atypical pattern (part-time, long hours, extra-long hours, nonstandard schedules, some weekends, most/all weekend), 11 combining two atypical patterns, and six combining three atypical patterns.

#### Covariates

Our study uses observational data and thus selection effects are an important threat to the interpretation of the findings. Workers are not randomly sorted into their work patterns. It is possible that characteristics associated with work hours or work schedules are also associated with sleep. To address this concern, it is important to control for factors that may be associated with the types of jobs individuals have and the nature of their work. These factors, which include demographics, socioeconomic position, health and lifestyle, and work conditions may also influence a person’s ability to both work and sleep [30, 45, 49–51]. Nonetheless, to help minimise the risk of both overcontrol and omitted variable bias, the selection of covariates was guided by purposeful selection methods i.e. a process of conducting univariate analyses with each covariate and outcome, followed by multivariate analyses in which the variables are added, fitted, deleted, and refitted [52].

Our demographic and socio-economic factors comprised of: gender (based on self-report: men, women), age and age-squared (because age may have a non-linear relationship with sleep [53]), marital status (single, married/cohabiting, separated/divorced/widowed), children in the household (youngest child aged 0–4 years, 5–9 years, 10–15 years), housing tenure (home-owner, private tenancy, public/social housing tenancy), informal caregiving for sick, disabled or elderly persons (none, co-resident, non-resident, both co-resident and non-resident), educational attainment (college degree or higher, A level [school-leaving qualification at 18 years], GCSE [school-leaving qualification at 16 years], other qualification, none), equivalised gross monthly household income (as quintiles), and National Statistics Socio-Economic Classification (NS-SEC – an indicator of social class determined by main job and employment status, based on differences in employment relations and occupational conditions: management/professional, intermediary, routine). As health problems that limit daily activity can affect workability [54] and sleep [55], we accounted for limiting long-term illness/disability (LLTI: no, yes). We included smoking status (never-smoker, ex-smoker, current smoker), because smoking is associated with work stress [56] and sleep [57]. We added frequency of alcohol consumption and physical exercise (each categorised as none, 1–2 times/week, 3–4 times/week, ≥ 5 times/week), because elevated alcohol consumption is associated with work-related stress [58], sleep inducement, and sleep disturbance [59, 60], whereas physical exercise may contribute to beneficial mental health [61] and sleep [62]. Sleep medication usage was added only as a sensitivity test, because results tend to be similar regardless of sleep medication use [63]. Although NS-SEC controls for some of the potential impact of employment relations and conditions, to further account for the psychosocial work environment, we created a second model adding the following work conditions: job satisfaction, leisure satisfaction, and income satisfaction (each categorised as satisfied, neutral satisfaction, dissatisfied); work autonomy—items on jobs task, work pace, work manner, task order, and work hours, summed and reverse coded (continuous range 1 [low autonomy] to 20 [high autonomy]); and physicality of the job (not at all, not very, fairly, very physical). When utilising our pooled sample, we accounted for the two response time periods by adjusting for wave (w4 and w7).

### Analyses

We assessed the distribution of sample characteristics and the prevalence of sleep outcomes with a descriptive analysis (with statistical differences tested by t-tests and regressions). To examine the relationships between each work pattern and sleep, we used multinomial logistic regression models for the categorical sleep duration outcome, and logistic regression models for the binary sleep disturbance outcome, and present odds ratios (OR). Separately, for each of the three exposure variables, the first model of our regression analyses adjusted for demographic, socioeconomic, and health factors. The second model added the work conditions so we could assess whether they explained any differences. To account for potential overlap between the work patterns, our third model included all three exposure variables. Data on nonstandard schedules and weekend working were not collected at wave 7, so model 3 used the w4-only sample, as did the supplementary testing where we analysed the 24 combinations of temporal work patterns (also adjusting for the covariates described for models 1 and 2).

We aggregated men and women in our main analyses. Although we were interested in examining gender differences, we gender-stratified only the sleep duration analyses for weekly work hours and weekend working, because we found only statistically significant gender interactions for predicting this outcome from these two patterns.

Survey weights were applied to all analyses to account for the complex design, sample attrition, and to generalize the results to the UK population. In the pooled sample, to correct for any autocorrelation (due to some individuals being interviewed at both waves) we clustered the analyses at the individual level. All analyses, performed in Stata V.15, used the MI commands and applied a significance level of 95% (*p* < 0.05).

## Results

### Descriptive characteristics

Table 1 reports descriptive statistics of all analysis variables. In both samples, two-fifths of workers experienced short sleep (< 7–8 h/night), few (< 4%) experienced long sleep (≥ 9 h/night), and 25% experienced sleep disturbance. Regarding work patterns, 31% worked part-time, more than a third worked longer than full-time (30% long hours and 8% extra-long hours), over half worked weekends (37% some and 22% most/all), and 28% worked nonstandard schedules. In examining sleep by work pattern, we find short sleep was most likely among people working extra-long hours (52%), long hours (44%), most/all weekends (45%), some weekends (43%), and nonstandard schedules (46%). Sleep disturbance was most likely among part-time workers (29%), most/all weekends (28%), and nonstandard schedules (29%).

**Table 1:** Descriptive statistics by temporal work pattern (by percentage unless otherwise stated)

	Pooled sample *n* = 48,990						w4-only sample *n* = 25,605							
		**Weekly work hours (hours/week)**^**a**^						**Weekend working**^**b**^				**Nonstandard schedules**^**b**^		
	**All**^**a**^	** < 35**	**35–40**	**41–54**	** ≥ 55**		**All**^**b**^	**None**	**Some**	**Most/All**		**Standard**	**Nonstandard**	
		*n* = 15,388	*n* = 17,422	*n* = 12,416	*n* = 3764			*n* = 10,853	*n* = 9251	*n* = 5501		*n* = 18,317	*n* = 7388	
	100	31.2	33.2	27.9	7.7		100	41.6	36.6	21.8		71.8	28.2	
**Sleep duration:**						***					***			***
< 7 h/night	41.6	39.2	39.6	43.8	52.0		41.4	38.2	42.7	45.0		39.4	46.2	
7–8 h/night	55.0	55.8	57.4	53.8	46.0		55.1	58.7	54.4	49.6		57.4	49.5	
≥ 9 h/night	3.4	5.0	3.0	2.4	2.0		3.5	3.1	2.9	5.4		3.2	4.3	
**Sleep disturbance:**						***					***			***
No disturbance	74.7	71.4	76.5	76.4	74.0		75.2	76.2	75.5	72.8		76.4	72.1	
Disturbance	25.3	28.6	23.5	23.6	26.0		24.8	23.8	24.5	27.2		23.6	27.9	
**Covariates**														
**Gender:**						***					***			***
Men	52.3	26.0	57.7	68.6	75.6		52.4	43.5	59.9	57.0		50.4	57.7	
Women	47.7	74.0	42.3	31.4	24.4		47.6	56.5	40.1	43.0		49.6	42.3	
**Age (years):**						***					***			***
Mean (SD)	42.6 (12.9)	44.6 (14.1)	41.5 (12.6)	41.6 (11.9)	42.6 (11.8)		42.4 (12.7)	43.3 (12.5)	42.7 (12.2)	40.2 (13.7)		42.8 (12.5)	41.4 (13.1)	
**Marital status:**						***					***			***
Single	23.2	21.2	26.7	21.8	20.9		21.6	19.8	19.5	28.8		20.5	24.6	
Married/cohabiting	68.7	68.5	65.4	71.8	72.5		69.9	71.2	72.4	63.2		70.9	67.4	
Separated/div/widow	8.1	10.2	7.9	6.4	6.6		8.5	9.0	8.1	8.0		8.7	8.0	
**Children in the household:**						***					ns			ns
None	62.2	56.7	66.6	63.0	63.0		62.1	62.3	61.1	63.3		62.3	61.6	
0–4 years	7.7	7.8	7.2	7.8	7.8		8.4	8.4	8.9	7.7		8.4	8.4	
5–11 years	15.4	18.2	13.1	15.3	15.3		14.8	14.5	15.5	14.1		14.7	15.0	
12–15 years	14.7	17.2	13.1	13.9	13.8		14.7	14.8	14.5	15.0		14.6	15.0	
**Housing tenure:**						***					***			***
Owner	72.6	71.6	72.6	74.1	72.1		73.4	75.9	74.8	66.3		75.1	69.1	
Private tenancy	14.8	13.0	15.4	15.1	18.1		14.9	13.3	15.0	17.9		14.2	16.6	
Public/social housing tenancy	12.6	15.4	12.0	10.8	9.8		11.7	10.8	10.2	15.8		10.7	14.3	
**Caregiving:**						***					ns			ns
None	85.2	81.1	86.4	87.8	87.5		84.7	84.4	84.9	85.0		84.9	84.4	
Co-resident	3.6	4.4	3.7	2.8	3.0		3.5	3.3	3.4	3.9		3.3	3.9	
Non-resident	10.6	13.8	9.4	9.1	8.8		11.3	11.8	11.1	10.4		11.2	11.3	
At both locations	0.6	8.7	0.6	0.3	0.7		5.5	0.5	0.5	0.7		0.6	0.4	
**Education attainment:**						***					***			***
Degree (or higher)	45.8	40.0	46.0	51.0	50.0		44.9	46.8	48.9	34.4		46.2	41.3	
A level (or equivalent)	23.0	22.3	24.3	22.8	20.9		22.6	21.3	21.7	26.4		21.8	24.5	
GCSE (or equivalent)	20.4	23.8	20.0	17.7	18.1		20.9	20.1	18.8	25.7		20.2	22.6	
Other qualification	7.0	8.4	6.4	5.9	7.7		7.4	7.4	7.0	8.2		7.6	7.0	
No qualification	3.8	5.5	3.3	2.6	3.3		4.2	4.4	3.5	5.3		4.1	4.5	
**NS-SEC occupation:**						***					***			***
Manager/professional	42.9	29.0	45.4	53.3	49.7		42.3	46.1	46.6	27.9		45.8	33.4	
Intermediate	23.4	27.0	25.8	16.8	23.0		23.7	25.1	22.1	23.6		24.4	21.8	
Routine	33.7	44.0	28.8	29.9	27.3		34.0	28.8	31.3	48.5		29.8	44.8	
**Equivalised household income:**						***					***			***
Quintile 5 (highest)	21.7	14.1	19.7	29.3	33.4		21.5	22.6	25.6	14.6		22.8	18.4	
Quintile 4	20.9	16.0	22.8	24.0	21.9		20.8	22.9	20.7	17.0		21.4	19.3	
Quintile 3	20.6	18.8	23.4	20.0	17.9		20.5	21.1	19.8	20.4		20.7	19.9	
Quintile 2	20.4	23.9	20.9	17.0	15.9		20.7	20.0	19.5	24.0		20.2	21.9	
Quintile 1 (lowest)	16.4	27.2	13.2	9.7	10.9		16.5	14.4	14.4	24.0		15.0	20.5	
**LLTI:**						***					ns			ns
No	75.7	72.2	76.8	77.6	77.5		75.6	74.8	76.6	75.2		75.8	75.0	
Yes	24.3	27.8	23.2	22.4	22.5		24.4	25.2	23.4	24.8		24.2	25.0	
**Smoker status:**						***					***			***
Non-smoker	45.6	46.3	47.0	44.1	42.2		43.8	46.9	42.5	39.9		44.6	41.5	
Ex-smoker	35.0	35.1	34.0	36.0	35.4		35.2	35.3	36.4	33.4		36.0	33.3	
Smoker	19.4	18.6	19.0	19.9	22.4		21.0	17.8	21.1	26.7		19.3	25.2	
**Exercise frequency:**						***					*			*
> 3 times/week	18.3	15.0	18.7	21.0	19.6		17.2	17.2	17.7	16.4		17.1	17.5	
1–3 times/week	22.9	21.8	22.3	24.5	23.5		22.9	23.0	23.5	21.6		23.6	21.1	
< 1 time/week	35.4	35.1	35.4	35.3	36.8		37.1	36.6	37.8	36.6		36.9	37.4	
No exercise	23.5	28.1	23.5	19.2	20.1		22.8	23.1	21.0	25.3		22.4	24.0	
**Alcohol consumption frequency:**						***					***			***
None	32.5	38.1	32.1	27.9	27.9		29.1	29.0	26.3	34.2		28.0	32.1	
1–2 days/week	35.6	33.6	36.6	36.4	37.1		37.3	38.0	35.6	38.6		37.2	37.6	
3–4 days/week	19.5	17.1	20.0	21.7	19.7		20.1	20.4	22.4	15.6		21.0	17.5	
≥ 5 days/week	12.3	11.2	11.3	14.0	15.3		13.5	12.6	15.7	11.6		13.8	12.8	
**Job satisfaction:**						***					ns			ns
Satisfied	79.0	80.2	77.3	79.0	81.4		77.6	77.1	78.6	76.9		77.9	76.9	
Neutral	8.8	8.3	9.4	9.0	7.6		9.3	9.5	8.9	9.7		9.0	9.9	
Dissatisfied	12.2	11.5	13.3	12.0	11.0		13.1	13.4	12.5	13.4		13.1	13.2	
**Income satisfaction:**						***					***			***
Satisfied	59.2	55.6	59.2	62.3	62.3		53.4	55.4	54.8	47.4		54.7	50.2	
Neutral	12.2	13.3	12.0	11.5	10.8		12.6	11.6	12.2	15.1		11.7	14.8	
Dissatisfied	28.7	31.1	28.8	26.2	26.9		34.0	33.0	33.0	37.5		33.6	35.0	
**Leisure satisfaction:**						***					***			***
Satisfied	54.3	60.6	56.4	49.2	38.7		51.6	55.4	51.5	44.2		53.0	47.7	
Neutral	14.0	14.4	14.2	13.8	12.5		14.4	14.3	13.4	16.2		14.2	15.0	
Dissatisfied	31.7	25.0	29.4	37.0	48.8		34.0	30.2	35.1	39.6		32.8	37.3	
**Job physicality:**						***					***			***
Not at all	22.6	23.4	20.5	22.7	27.5		22.7	16.5	22.9	34.5		20.4	28.7	
Not very	38.1	41.2	35.5	37.4	39.6		38.2	34.9	39.0	43.4		36.2	43.4	
Fairly	25.2	23.0	26.9	26.3	23.0		25.4	30.4	25.6	15.4		27.8	19.3	
Very	14.1	12.4	17.1	13.6	9.9		13.7	18.2	12.5	6.7		15.6	8.5	
**Work autonomy:**						***					***			***
Mean (SD)	11.4 (4.1)	10.8 (4.3)	11.4 (3.8)	11.9 (3.8)	12.4 (3.9)		11.4 (4.1)	11.2 (3.9)	11.7 (4.0)	11.1 (4.3)		11.6 (3.9)	10.8 (4.3)	

Data are multiply imputed. Sample sizes are unweighted. Survey weights were applied to determine percentages and means

^a^ The analytic sample for weekly work hours used the pooled data, with analyses clustered at the individual level

^b^The analytic sample for weekend working and nonstandard schedules used w4-only data

^*^p < 0.05 indicate statistically significant differences between categories within each work pattern

^**^p < 0.01

^***^p < 0.001

In both pooled and w4-only samples, there were more men (52%) than women. Whilst part-time work was more prevalent among women, long hours, weekends, and nonstandard schedules were more prevalent among men. Most were married (70%), homeowners (73%), had no children (62%), provided no informal caregiving (85%), had no LLTI (76%), and were satisfied with their jobs (77%-78%), incomes (53%-59%) and leisure (52–54%). Marriage, home-ownership, college degrees, and professional/managerial occupations were common among individuals working longer than full-time. Part-time work was associated with caregiving and LLTI. Being single, rented housing (private and social), and routine occupations were associated with weekend working and nonstandard schedules. Individuals working extra-long hours, weekends, and non-standard schedules were the most likely to smoke, and frequently consume alcohol. As weekly work hours increased, so did work autonomy and income satisfaction, but leisure satisfaction declined. Leisure satisfaction, income satisfaction and work autonomy were also lower for individuals working weekends (compared to non-weekends) and nonstandard schedules (compared to standard schedules).

### Temporal work patterns and sleep

#### Sleep duration

In Table 2, we present regression models examining the association between temporal work patterns and sleep. First, in panel A, we provide results for sleep duration. Compared to full-time hours, part-time workers had higher odds of long sleep (OR = 1.40), whereas individuals working longer hours had higher odds of short sleep (long hours OR = 1.20, extra-long hours OR = 1.65) (Model 1). In Model 3 (utilising the w4-only sample), which adjusted for nonstandard schedules and weekend working, part-time working remained associated with long sleep (OR = 1.29), and longer hours with short sleep (long hours OR = 1.11, extra-long hours OR = 1.39).

**Table 2:** Associations between temporal work patterns and sleep duration^a^, and sleep disturbance^b^

**Panel A**					**Sleep duration (ref: 7–8 h/night)**							
	**Model 1**				**Model 2**				**Model 3**			
	< 7 h/night		≥ 9 h/night		< 7 h/night		≥ 9 h/night		< 7 h/night		≥ 9 h/night	
**Temporal work patterns**	OR	95% CI	OR	95% CI	OR	95% CI	OR	95% CI	OR	95% CI	OR	95% CI
**Weekly work hours (ref: 35–40 h/wk)**^**c**^	1.00		1.00		1.00		1.00		1.00		1.00	
< 35 h/wk (part-time)	0.91	0.84, 0.97	1.40	1.16, 1.69	0.94	0.87, 1.01	1.41	1.16, 1.70	0.94	0.86, 1.03	1.29	1.00, 1.67
41–54 h/wk (long hours)	1.20	1.12, 1.29	0.97	0.78, 1.21	1.17	1.10, 1.26	0.97	0.78, 1.21	1.11	1.01, 1.22	0.86	0.64, 1.15
≥ 55 h/wk (extra-long hours)	1.65	1.48, 1.84	1.03	0.72, 1.48	1.56	1.39, 1.74	1.02	0.71, 1.47	1.39	1.20, 1.61	0.79	0.50, 1.25
**Weekend working (ref: no weekends)**^**d**^	1.00		1.00		1.00		1.00		1.00		1.00	
Some weekends	1.19	1.10, 1.28	1.05	0.83, 1.33	1.18	1.09, 1.27	1.04	0.82, 1.31	1.09	1.00, 1.18	1.02	0.80, 1.31
Most/all weekends	1.36	1.24, 1.49	1.52	1.21, 1.92	1.30	1.18, 1.43	1.50	1.18, 1.89	1.13	1.02, 1.25	1.45	1.11, 1.89
**Schedules (ref: standard schedules)**^**d**^	1.00		1.00		1.00		1.00		1.00		1.00	
Nonstandard	1.31	1.22, 1.42	1.28	1.04, 1.57	1.28	1.19, 1.39	1.26	1.02, 1.54	1.19	1.10, 1.30	1.13	0.90, 1.42
**Panel B**					**Sleep disturbance (ref: no disturbance)**							
**Temporal work patterns**	**Model 1**				**Model 2**				**Model 3**			
	OR		95% CI			OR	95% CI		OR		95% CI	
**Weekly work hours (ref: 35–40 h/wk)**^**c**^	1.00				1.00				1.00			
< 35 h/wk (part-time)	1.04		0.96, 1.13		1.10		1.01, 1.19		1.04		0.94, 1.15	
41–54 h/wk (long hours)	1.11		1.02, 1.20		1.07		0.99, 1.16		1.03		0.93, 1.14	
≥ 55 h/wk (extra-long hours)	1.30		1.16, 1.47		1.20		1.07, 1.36		1.00		0.85, 1.18	
**Weekend working (ref: no weekends)**^**d**^	1.00				1.00				1.00			
Some weekends	1.14		1.04, 1.24		1.12		1.02, 1.22		1.07		0.97, 1.17	
Most/all weekends	1.21		1.10, 1.34		1.14		1.03, 1.26		1.05		0.94, 1.18	
**Schedules (ref: standard schedules)**^**d**^	1.00				1.00				1.00			
Nonstandard schedules	1.24		1.15, 1.35		1.22		1.12, 1.33		1.20		1.09, 1.31	

Data are multiply imputed. Sample sizes are unweighted. Survey weights were applied in regression analyses

^a^multinomial logistic regression models

^b^logistic regression models

^c^Weekly work hours – models 1 & 2 analysed the pooled sample data (*n* = 48,990) and the analyses were clustered at the individual level; model 3 analysed the w4-only sample data (*n* = 25,605)

^d^Weekend working and nonstandard schedules analysed the w4-only sample data (*n* = 25,605)

Model 1 analysed the associations for each type of temporal work pattern (e.g. weekly work hours only; weekend working only; or schedules only), and adjusted only for gender, age, age-squared, marital status, youngest child in the household, informal caregiving, housing tenure, educational attainment, equivalised household income, NS-SEC, LLTI, smoker status, exercise frequency, and frequency of alcohol consumption

Model 2 = model 1 + work conditions: job satisfaction, satisfaction with income, satisfaction with leisure time, work autonomy, and job physicality

Model 3 = model 2 + all temporal work patterns (weekly work hours, weekend working and schedules)

Weekend workers had greater odds of short sleep than non-weekend workers, with the highest frequency of weekend working associated with the highest odds (some weekends OR = 1.19, most/all weekends OR = 1.36) (Model 1). Working most/all weekends was also associated with higher odds of long sleep (OR = 1.52) (Model 1). Adjustment for weekly work hours and nonstandard schedules attenuated these relationships a little (Model 3) suggesting some overlap between temporal work patterns. Relative to standard schedules, workers with nonstandard schedules had higher odds of short sleep (OR = 1.31) and of long sleep (OR = 1.28) (Model 1). Adjustment for weekly work hours and weekend working reduced these differences, and the association with long sleep lost statistical significance at the 5% level (Model 3).

#### Sleep disturbance

In panel B we present logistic regressions predicting sleep disturbance. Working longer than 35–40 h/week was associated with higher odds of sleep disturbance (long hours OR = 1.11, extra-long hours OR = 1.30) (Model 1). After adjustment for work conditions (Model 2), the odds attenuated for extra-long hours (OR = 1.20), and lost statistical significance at the 5% level for long hours, but part-time hours had higher odds of disturbance (OR = 1.10). This may reflect higher job satisfaction, income satisfaction and work autonomy among individuals working longer hours, and lower work autonomy and more income dissatisfaction among individuals working fewer hours. After adjustment for weekend working and nonstandard schedules (Model 3 using the w4-only sample), there were no significant associations between weekly work hours and sleep disturbance.

Weekend workers had higher odds of sleep disturbance than non-weekend workers. The odds increased as the frequency of weekend working increased (some weekends OR = 1.14, most/all OR = 1.21) (Model 1). The odds for most/all weekends attenuated (OR = 1.14) after accounting for work conditions (Model 2), which may be explained by higher rates of dissatisfaction with income and leisure. After adjustment for weekly work hours and nonstandard schedules, there was no apparent association between weekend working and sleep disturbance (Model 3).

Lastly, individuals working nonstandard schedules had higher odds of sleep disturbance than standard schedules (OR = 1.24) (Model 1). Adjustment for work conditions (Model 2), weekly work hours, and weekend working (Model 3) did not change this association.

#### Gender-stratified results

The gender-stratified regression results presented in Table 3 (for the two work patterns which had shown statistically significant gender interactions with sleep duration) suggests there are gender differences in sleep duration related to weekly work hours and weekend working. Compared to working 35–40 h/week, only men who worked part-time had higher odds of long sleep (OR = 1.68), whilst both men and women had higher odds of short sleep if working long hours (men OR = 1.12, women 1.25) or extra-long hours (men OR = 1.58 for men, women OR = 1.68) (Model 1). After adjusting for work conditions (Model 2), the gender difference for extra-long hours disappeared (men OR = 1.54, women OR = 1.50), possibly reflecting greater leisure dissatisfaction and lower work autonomy among women working extra-long hours (as shown in  Additional file 2, a table of the gender-stratified sample characteristics).

**Table 3:** Associations between temporal work patterns and sleep duration^a^ in a gender-stratified sample of workers

	**Men**									**Women**								
	**Sleep duration (ref: 7–8 h/night)**									**Sleep duration (ref: 7–8 h/night)**								
		**Model 1**				**Model 2**					**Model 1**				**Model 2**			
		** < 7 h/night**		** ≥ 9 h/night**		** < 7 h/night**		** ≥ 9 h/night**			** < 7 h/night**		** ≥ 9 h/night**		** < 7 h/night**		** ≥ 9 h/night**	
**Temporal work patterns**	**%**	**OR**	**95% CI**	**OR**	**95% CI**	**OR**	**95% CI**	**OR**	**95% CI**	**%**	**OR**	**95% CI**	**OR**	**95% CI**	**OR**	**95% CI**	**OR**	**95% CI**
**Weekly work hours (ref: 35–40 h/wk)**^**b**^	36.8	1.00		1.00		1.00		1.00		29.4	1.00		1.00		1.00		1.00	
< 35 h/wk	15.3	0.90	0.77, 1.06	1.68	1.11, 2.56	0.94	0.80, 1.10	1.71	1.13, 2.61	48.5	0.94	0.84, 1.06	1.20	0.87, 1.65	0.97	0.86, 1.09	1.19	0.86, 1.64
41–54 h/wk	36.7	1.12	1.00, 1.26	0.99	0.64, 1.54	1.12	1.00, 1.26	0.96	0.62, 1.49	18.3	1.25	1.09, 1.44	0.89	0.61, 1.31	1.20	1.04, 1.38	0.88	0.60, 1.30
≥ 55 h/wk	11.2	1.58	1.34, 1.86	1.01	0.57, 1.79	1.54	1.30, 1.81	0.93	0.52, 1.68	3.9	1.68	1.31, 2.17	1.01	0.48, 2.11	1.50	1.16, 1.94	0.93	0.44, 1.97
**Weekend working (ref: no weekends)**^**c**^	34.6	1.00		1.00		1.00		1.00		49.5	1.00		1.00		1.00		1.00	
Some weekends	41.7	1.07	0.96, 1.20	1.03	0.69, 1.52	1.07	0.96, 1.20	1.00	0.68, 1.46	30.8	1.34	1.20, 1.49	1.09	0.81, 1.47	1.31	1.18, 1.46	1.08	0.80, 1.45
Most/all weekends	23.7	1.26	1.10, 1.44	1.56	1.05, 2.32	1.22	1.06, 1.40	1.51	1.02, 2.25	19.8	1.48	1.30, 1.68	1.52	1.15, 2.02	1.38	1.21, 1.57	1.48	1.11, 1.97

^a^Multinomial logistic regression models

^b^Weekly work hours – model 1 analysed the pooled sample data (*n* = 48,990) and the analyses were clustered at the individual level; model 2 analysed the w4-only sample data (*n* = 25,605)

^c^Weekend working analysed the w4-only sample data (*n* = 25,605)

Model 1 analysed the associations for each type of temporal work pattern (e.g. weekly work hours only; weekend working only; or schedules only), and adjusted for age, age-squared, marital status, youngest child in the household, informal caregiving, housing tenure, educational attainment, equivalised household income, NS-SEC, LLTI, smoker status, exercise frequency, and frequency of alcohol consumption

Model 2 = model 1 + work conditions: job satisfaction, satisfaction with income, satisfaction with leisure time, work autonomy, and job physicality

Relative to non-weekends, men had greater odds (OR = 1.26) of short sleep if they worked most/all weekends (Model 1). Women who worked any weekends had higher odds of short sleep (some weekends OR = 1.34, most/all weekends OR = 1.48) (Model 1), though the odds for most/all weekends attenuated (OR = 1.38) upon adjustment for work conditions (Model 2). Working most/all weekends was also associated with long sleep, but there was no gender difference for this association.

### Supplementary tests

#### Overlap between work patterns

Our main results suggested the relationships between the atypical work patterns and sleep weakened upon adjusting for other temporal work patterns. To further examine overlapping work patterns, we analysed the work patterns as 24 separate categories. Additional file 3 shows that whilst 17% of our w4-only sample worked no atypical temporal patterns (the reference category), 33% worked one pattern (panel A), 31% worked two (panel B), and 18% worked three (panel C).

Twenty-four is a large number of categories, and some categories have a low prevalence, including panel A’s extra-long hours only (0.7%), and panel B’s extra-long hours combined with nonstandard schedules (0.2%). This gives rise to uncertainty in our regression results and the possibility of type I errors. Nonetheless, the results in panel A are similar to those from our main analysis for sleep duration - working longer than 35–40 h/week, frequent weekend working, and nonstandard schedules are each associated with short sleep. Of the categories which combined two atypical patterns (panel B), 73% were associated with short sleep, with the highest odds of short sleep associated with combinations which included working long/extra-long hours (e.g. ORs ranged from 2.00 to 2.88). Of the categories which combined three atypical patterns (panel C), 61% were associated with short sleep. The combination extra-long hours with both nonstandard schedules and most/all weekends had the highest odds (OR = 1.97).

The associations between sleep disturbance and individual atypical work patterns (panel A) were similar in magnitude to those in our main analysis, but they were not statistically significant at the 5% level. However, compared to the reference category (no atypical patterns), 45% of the categories in panel B (combinations of two atypical patterns) had higher odds of sleep disturbance. The highest odds were for combinations that included nonstandard schedules. Of the categories in panel C (combinations of three atypical patterns), 83% were associated with sleep disturbance. Of these, long hours combined with some weekends and nonstandard schedules had the highest odds of sleep disturbance (OR = 1.51).

#### Sleep medication

Few (< 6%) of our sample used sleep medication, though usage was more likely among workers with part-time hours compared to other work patterns, and those who had a sleep disturbance. However, our regression results (available on request) which adjusted for usage did not differ substantially to the main results.

## Discussion

We found atypical temporal work patterns were associated with poor sleep in a nationally representative sample of working people in the UK. Sleep duration was inversely related to working hours, with long and extra-long hours associated with short sleep, and part-time hours associated with long sleep. Weekend working and nonstandard work schedules were associated with both short and long sleep. All atypical weekly work hours, weekend working and nonstandard schedules related to more sleep disturbance.

These results, generalisable to the UK workforce, were based on household panel data, representing a diverse sample of all workers aged 16 + , including women. They build on previous research which mainly concentrated on workers in particular occupational settings, and/or from Scandinavia and East Asia, who were engaged in long weekly hours or shiftwork, and which mostly measured only insufficient or problematic sleep.

Our finding that long working hours were related to short sleep in an aggregated sample of men and women extends research which found similar associations among male occupational cohorts [30]. Consistent with results from a Spanish health survey [64], our gender-stratified analysis suggested this association was stronger for women than men. Our results might be explained by the hypothesis that the demands of long working hours stimulate physiological arousal and hinder sleep [65]. Likewise, they could be due to scarcity theory - the more time allocated for work, the more truncated sleep could be [23]. This may be especially pertinent to women, who tend to provide more informal caregiving and domestic labour than their partners [66, 67], thus increasing their total (paid plus unpaid) working hours. Nonetheless, adjustment for work conditions such as leisure satisfaction and work autonomy narrowed these gender differences, which suggests improving women’s working conditions could be important in reducing gender inequalities in sleep.

We also found part-time workers were more likely to experience longer sleep than full-time workers. Our gender-stratified analysis suggested this was only true for men. It is noteworthy that women tend to work part-time to combine work and family duties [68], whereas men tend to do so due to under-employment [69]. Under-employment is associated with depression [70], which relates to both short and long sleep, though long sleep is more likely [71].

Previous research has related one type of nonstandard schedule (shiftwork) to insufficient sleep [28]. Similarly, we found nonstandard work (schedules and weekends) related to short sleep. Moreover, our multinomial regression analyses enabled us to note that it also related to long sleep. Of the few studies investigating long sleep, long sleep was associated with shiftwork among Finnish hospital employees, as was fatigue—indicating a high need for recovery [72]. There is no comparable study on weekend working and sleep, but it is noteworthy that there is substantial diversity in nonstandard work (e.g. time demands, scheduling control, and rotations) which differ in the amount of time available for recovery. It follows then, some nonstandard workers may sleep longer due to fatigue, and others sleep less due to time constraints. Other than weekend working, our analyses did not differentiate between types of nonstandard work, but future research could do so.

Based on nine studies on shiftwork and working hours, mostly from Scandinavian public sector workers, a review concluded that work scheduling impacts sleep disturbance [29]. Consistent with this, we found disturbance was highest among workers with atypical temporal patterns. Long and irregular work hours contribute to work-life interference, which in turn contributes to circadian rhythm disruption and stress, which interfere with sleep [73]. Long work hours, weekend working, and early morning work (particularly among later chronotypes ‘night owls’) have also been associated with depressive symptoms, and depressive symptoms with sleep interference [74, 75]. Furthermore, as noted when we adjusted for work conditions such as leisure satisfaction, poor psychosocial work factors, which negatively impact sleep [29], may partially explain our findings. A lack of relaxation time could be a causal pathway between long work hours and sleep [45]. The cross-sectional design of our investigation did not support mediational analysis, but future research might consider this.

A strength of our study is our consideration of the two fundamental components of sleep – quantity and quality [76]. However, whilst we found that most of the atypical work patterns were associated with both short sleep and sleep disturbance, the inter-relatedness of sleep duration and sleep disturbance should be recognised. Individuals who experience problems falling and staying asleep could find their sleep is truncated. These individuals could deem their overall sleep quality as poor. Future research could explore this inter-relatedness, particularly as there are suggestions that sleep quality may be more important than duration in predicting future health [76].

Future research, such as cluster analysis techniques, could also help identify the similarities between atypical temporal work patterns. The present study only mutually adjusted for all three exposure variables, but our finding that the strength of the relationships between the work patterns and sleep reduced or lost statistical significance, suggested some overlap between the patterns. Supplementary testing enabled us to further investigate this. Our results indicated that 49% of workers had overlapping patterns, and these were associated with the poorest sleep – especially when workers combined long/extra-long hours or nonstandard schedules with at least one other pattern. However, due to our large number of categories (i.e. 24) and the low prevalence of some work pattern combinations (e.g. 0.2% extra-long hours combined with non-standard schedules), these results could include type I error. Furthermore, we subjected our dataset to multiple testing, which increases the probability of a false-positive finding. As a result we are careful to only report consistent findings [77]. A further limitation is that although we accounted for factors that may influence a person’s ability to both work and sleep, some covariates may be mediators rather than confounders, and we may therefore be over-adjusting.

Other limitations of our work include the use of self-reported data – which can be subject to biases. Although our sleep measures were derived from the psychometrically validated PSQI, the UKHLS does not administer all 19 PSQI items and provides no validated scoring system for them. Furthermore, participants were only asked about their usual sleep habits during the past month, they were not asked to differentiate between sleep on week days and weekends or workdays and free days. Yet, we know that some individuals experience work-related stresses that can interfere with workday sleep, and that workers sleep in on weekends/non-work days to compensate for induced sleep debt [10]. Nonetheless, research suggests the PSQI reveals sleep habits on workdays [74].

Extant studies guided our use of the PSQI items, notwithstanding, our results may not be directly comparable to other studies. Nonetheless, participants were asked to record how much sleep they achieved, which may be a more accurate measure of sleep compared to studies which ask respondents how much time they spent in bed. Furthermore, to be consistent with contemporary recommendations about sleep duration [78], we replaced the original PSQI upper category (> 7 h/night) with ≥ 9 h/night, and introduced a reference category of 7–8 h/night. Nevertheless, people’s sleep needs differ, e.g., naturally short sleepers feel well-rested after 4–6 h sleep/night [79]. Therefore, in future, deviation from participants’ own optimal sleep habits might be a more useful measure [1].

Furthermore, as overall sleep patterns may be more critical to long-term health than snapshots in time [2], future data collections may enable us to analyse change over time and assess longitudinal associations. Although some of our analysis used pooled waves of data, enabling us to measure work and sleep across two time points spanning three years, our study was cross-sectional in design. Accordingly, we cannot rule out the possibility of reverse causation due to pre-existing sleep problems, nor claim a causal relationship between temporal work patterns and sleep.

## Conclusion

Our study shows a link between atypical temporal work patterns and poor sleep in a heterogeneous UK population. We found the poorest sleep among individuals working extra-long hours, frequent weekends, and nonstandard schedules (worked in isolation and in combination). Considering the economic and health costs of poor sleep, employers and policymakers should put to bed the erroneous notion that sleep is a waste of time and, instead, take steps to help workers achieve a good night’s sleep. Workers should be involved in setting shift rotations, ensuring they have some work-free evenings. Work schedules could be matched to their chronotypes (‘larks’ and ‘owls’). Employers should monitor working hours, assess workloads, and ensure workers have sufficient breaks and rest periods. They should judge workers on their output rather than their presence in the workplace, tackle the culture of overtime work environments (where people are expected to arrive early and leave late), and heed their legal obligations to reduce work-related stress. Also, they should consider compensating individuals who are required to work long and irregular hours. Future research could investigate what kind of, and how much, compensation would be sufficient to offset the negative consequences of these work patterns.

Policymakers should act to create a more inclusive society considering the biological and social needs of people working outside the usual 9–5. A starting point might be to ensure employers include psychosocial risks in their health & safety assessments. They might also follow France’s example and legislate to give workers the right to disconnect from work in their downtime.

### Supplementary Information

**Additional file 1: Table A1.** Missing data and the use of auxiliary variables in imputations. This is a table with an explanation regarding the amount of missing data and the imputation modelling.

**Additional file 2: Table A2.** Gender-stratified descriptive statistics by weekly work hours and by weekend working^a^. This is a table showing the gender-stratified descriptive statistics.

**Additional file 3: Table A3.** Associations between combinations of temporal work patterns and sleep duration^a^, and sleep disturbance^b^. This is a table showing the regression results for the associations between different combinations of temporal work patterns and sleep.

## Data Availability

The UKHLS dataset is available under End User Licence from the UK Data Archive https://discover.ukdataservice.ac.uk/catalogue/?sn=6614 Data documentation is available from the Understanding Society website https://www.understandingsociety.ac.uk/documentation.

## Abbreviations

CI: Confidence interval
DSM-V: The Diagnostic and Statistical Manual of Mental Disorders, Fifth Edition
GCSE: General certificate of secondary education
LLTI: Limiting long-term illness/disability
MICE: Multiple imputation by chained equation
NS-SEC: National Statistics Socio-Economic Classification
OR: Odds ratio
PSQI: Pittsburgh Sleep Quality Index
UK: United Kingdom
UKHLS: UK Household Longitudinal Study
W4: Wave 4
W7: Wave 7
