# Supplementary material for: Work hours, weekend working, nonstandard work schedules and sleep quantity and quality: findings from the UK household longitudinal study
Source: BMC Public Health. 2024 Jan 27;24:309. doi: 10.1186/s12889-024-17762-0 (PMC10821573; doi:10.1186/s12889-024-17762-0)
Supplement: Supplementary file 1 — Additional file 1: Table A1. Missing data and the use of auxiliary variables in imputations. This is a table with an explanation regarding the amount of missing data and the imputation modelling. [file 12889_2024_17762_MOESM1_ESM.docx]

Additional file 1.docx

Table A1 Missing data and the use of auxiliary variables in imputations

|  | **Pooled wave 4 & 7 sample**  **n=48,990** | **Wave 4 only sample**  **n=25,605** |
| --- | --- | --- |
|  | **% missing** | **% missing** |
| **Exposures** |  |  |
| Weekly work hours ^a^ | 2.0 | 1.4 |
| Weekend working ^a^ | n/a | 10.1 |
| Nonstandard schedules ^a^ | n/a | 10.1 |
| **Outcomes** |  |  |
| Sleep duration ^b^ | 8.6 | 9.3 |
| Sleep latency ^b^ | 8.4 | 9.3 |
| Sleep maintenance ^b^ | 8.4 | 9.3 |
| Sleep quality ^b^ | 8.4 | 9.3 |
| **Covariates** |  |  |
| Gender | <0.01 | 0.0 |
| Age | <0.01 | 0.0 |
| Marital status | 0.1 | 0.1 |
| Children in the household | 0.4 | 0.01 |
| Housing tenure | 1.1 | 0.9 |
| Caring location | 0.04 | 0.04 |
| Caring intensity ^c^ | 0.4 | 0.4 |
| Education attainment | 1.2 | 0.3 |
| NS-SEC occupation ^g^ | 2.3 | 1.9 |
| Equivalised household income | 0.6 | 0.03 |
| Long-term illness or disability | 0.1 | 0.1 |
| Smoker status | 20.8 | 18.1 |
| Exercise frequency | 21.4 | 18.4 |
| Alcohol consumption frequency | 28.0 | 27.7 |
| **Potential Mediators** |  |  |
| Job satisfaction | 9.5 | 10.2 |
| Income satisfaction | 13.1 | 16.1 |
| Leisure satisfaction | 13.1 | 16.1 |
| Job physicality | 7.3 | 25.3 |
| Work autonomy | 18.5 | 10.2 |
| **Additional auxiliary variables** |  |  |
| Contract (permanent/temporary) ^c^ | 1.3 | 1.1 |
| Employment (employee/self-employed) ^c^ | 1.2 | 1.1 |
| Workplace (on-site/remote) ^c^ | 1.2 | 1.0 |
| Sleep cough ^c^ | 8.9 | 10.0 |
| Sleep fatigue ^c^ | 8.4 | 9.3 |
| sleep medication ^d^ | 8.4 | 9.3 |
| Lagged (w6 and/or w3) NS-SEC occupation ^e^ | 17.1% | 16.8% |
| **Overall missingness** | 44.0 | 46.0 |

a when not used in a model as an exposure, acted as an auxiliary variable in the imputation model for other exposures; b when not used in a model as an outcome, acted as an auxiliary variable in the imputation model. c used only as auxiliary variables. d when not used in a sensitivity analysis, acted as an auxiliary variable in the imputation model. ^e^ also added in NS-SEC at the previous wave as an auxiliary variable.

The data including in the modelling (exposures, outcomes, covariates, auxiliary variables and design weights), were multiply imputed by chained equations (MICE) in STATA V.15 and 46 datasets were generated for each analytic sample. The number of imputations was determined by applying the ‘rule of thumb’ recommendation to match them to the total amount of missingness in the samples (i.e., 46% overall missingness=46 imputed datasets). The imputation diagnostics (e.g., Monte Carlo errors, relative increase in variance, frequency of missing information, relative efficiency) were checked to ensure that the number of imputations was sufficient and the imputation model reasonable [1].

## References

1 White IR, Royston P, Wood AM. Multiple imputation using chained equations: Issues and guidance for practice. *Stat Med* 2011;**30**:377–99. doi:10.1002/sim.4067
