# Supplementary material for: Work hours, weekend working, nonstandard work schedules and sleep quantity and quality: findings from the UK household longitudinal study
Source: BMC Public Health. 2024 Jan 27;24:309. doi: 10.1186/s12889-024-17762-0 (PMC10821573; doi:10.1186/s12889-024-17762-0)
Supplement: Supplementary file 2 — Additional file 2: Table A2. Gender-stratified descriptive statistics by weekly work hours and by weekend workinga. This is a table showing the gender-stratified descriptive statistics. [file 12889_2024_17762_MOESM2_ESM.docx]

Additional file 2.docx

Table A2 Gender-stratified descriptive statistics by weekly work hours and by weekend working^a^

|  | **Men (n=24724)^b^** | | | | | | **Men (n=12992)^c^** | | | | | **Women (n=24265)^b^** | | | | | | **Women (n=1213)^c^** | | | | |
| --- | --- | --- | --- | --- | --- | --- | --- | --- | --- | --- | --- | --- | --- | --- | --- | --- | --- | --- | --- | --- | --- | --- |
|  | **All** | **Weekly work hours (hr/wk)** | | | | | **All** | **Weekend working** | | | | **All** | **Weekly work hours (hr/wk)** | | | | | **All** | **Weekend working** | | | |
|  | **Men** | **<35**  **n= 3908** | **35-40**  **n= 9876** | **41-54**  **n=**  **8144** | **≥55**  **n= 2796** |  | **Men** | **None**  **n=**  **4532** | **Some**  **n= 5430** | **Most/**  **All**  **n=**  **3030** |  | **Women** | **<35**  **n=**  **11489** | **35-40 n= 7506** | **41-54**  **n= 4304** | **≥55**  **n= 966** |  | **Women** | **None**  **n= 6300** | **Some**  **n= 3877** | **Most/**  **All**  **n= 2436** |  |
|  | 100 | 15.3 | 36.8 | 36.7 | 11.2 |  | 100 | 34.6 | 41.7 | 23.7 |  | 100 | 48.5 | 29.4 | 18.3 | 3.9 |  | 100 | 49.5 | 30.8 | 19.8 |  |
| **Sleep duration:** |  |  |  |  |  | *** |  |  |  |  | *** |  |  |  |  |  | *** |  |  |  |  | *** |
| <7 hours/night | 43.4 | 39.2 | 41.1 | 44.6 | 52.8 |  | 43.5 | 44.5 | 43.5 | 46.3 |  | 39.7 | 39.3 | 37.6 | 41.9 | 50.3 |  | 38.9 | 35.6 | 41.6 | 42.9 |  |
| 7-8 hours/night | 54.2 | 55.8 | 56.8 | 53.6 | 45.6 |  | 53.8 | 56.3 | 54.3 | 49.4 |  | 55.9 | 55.8 | 58.1 | 54.6 | 46.7 |  | 56.6 | 60.5 | 54.6 | 50.1 |  |
| ≥9 hours/night | 2.4 | 5.0 | 2.1 | 1.2 | 1.6 |  | 2.7 | 2.2 | 2.2 | 4.3 |  | 4.4 | 4.9 | 4.3 | 3.5 | 3.0 |  | 4.5 | 3.9 | 3.8 | 7.0 |  |
|  |  |  |  |  |  |  |  |  |  |  |  |  |  |  |  |  |  |  |  |  |  |  |
| **Covariates** |  |  |  |  |  |  |  |  |  |  |  |  |  |  |  |  |  |  |  |  |  |  |
| **Age (years):** |  |  |  |  |  | *** |  |  |  |  | *** |  |  |  |  |  | *** |  |  |  |  | *** |
| Mean (SD) | 42.4  (13.2) | 45.5  (17.3) | 41.8  (12.7) | 41.7  (11.9) | 42.8  (11.8) |  | 42.3  (13.0) | 43.2  (13.1) | 42.6  (12.2) | 40.3  13.9) |  | 42.7  (12.6) | 44.3  (12.8) | 41.1  (12.5) | 41.1  (11.9) | 42.2  (11.9) |  | 42.5  (12.4) | 43.4  (12.1) | 42.8  (12.0) | 40.0  13.5) |  |
| **Marital status:** |  |  |  |  |  | *** |  |  |  |  | *** |  |  |  |  |  | *** |  |  |  |  | *** |
| Single | 24.1 | 32.0 | 26.1 | 20.3 | 19.0 |  | 22.1 | 22.0 | 18.8 | 28.2 |  | 22.2 | 17.5 | 27.6 | 25.4 | 26.6 |  | 21.1 | 18.1 | 20.5 | 29.5 |  |
| Married/cohabiting | 71.1 | 62.1 | 69.1 | 75.2 | 76.5 |  | 72.7 | 73.1 | 75.8 | 66.8 |  | 66.1 | 70.7 | 60.3 | 64.2 | 60.3 |  | 66.8 | 69.7 | 67.4 | 58.4 |  |
| Separated/div/widow | 4.8 | 5.9 | 4.8 | 4.5 | 4.5 |  | 5.2 | 4.9 | 5.4 | 5.0 |  | 11.7 | 11.8 | 12.1 | 10.4 | 13.1 |  | 12.1 | 12.2 | 12.1 | 12.1 |  |
| **Children in the household:** |  |  |  |  |  | *** |  |  |  |  | ** |  |  |  |  |  | *** |  |  |  |  | * |
| None | 62.3 | 72.3 | 63.0 | 58.4 | 59.2 |  | 61.6 | 63.9 | 59.2 | 62.5 |  | 62.2 | 51.2 | 71.6 | 73.3 | 74.0 |  | 62.6 | 61.0 | 63.9 | 64.3 |  |
| 0-4 years | 8.5 | 5.1 | 8.8 | 9.4 | 9.3 |  | 9.4 | 9.3 | 10.1 | 8.6 |  | 6.7 | 8.8 | 5.1 | 4.3 | 4.3 |  | 7.3 | 7.8 | 7.1 | 6.4 |  |
| 5-11 years | 15.3 | 10.7 | 14.5 | 17.5 | 16.7 |  | 15.1 | 14.0 | 16.1 | 14.6 |  | 15.7 | 20.8 | 11.0 | 10.4 | 11.0 |  | 14.5 | 14.9 | 14.6 | 13.3 |  |
| 12-15 years | 13.9 | 11.9 | 13.7 | 14.7 | 14.8 |  | 13.9 | 12.8 | 14.6 | 14.3 |  | 15.4 | 19.1 | 12.2 | 11.9 | 10.7 |  | 15.6 | 16.2 | 14.4 | 16.0 |  |
| **Housing tenure:** |  |  |  |  |  | *** |  |  |  |  | *** |  |  |  |  |  | *** |  |  |  |  | *** |
| Home-owner | 72.8 | 69.6 | 73.1 | 73.9 | 72.3 |  | 73.5 | 75.8 | 75.2 | 67.3 |  | 72.5 | 72.2 | 71.9 | 74.4 | 71.6 |  | 73.3 | 76.0 | 74.2 | 65.1 |  |
| Private tenancy | 15.4 | 14.2 | 15.1 | 15.2 | 18.4 |  | 15.8 | 14.1 | 15.2 | 19.0 |  | 14.1 | 12.6 | 15.7 | 14.9 | 17.4 |  | 13.9 | 12.5 | 14.6 | 16.4 |  |
| Public/social housing tenancy | 11.8 | 16.2 | 11.7 | 10.9 | 9.4 |  | 10.7 | 10.1 | 9.6 | 13.7 |  | 13.4 | 15.1 | 12.4 | 10.7 | 11.1 |  | 12.8 | 11.5 | 11.2 | 18.5 |  |
| **Caregiving:** |  |  |  |  |  | *** |  |  |  |  | * |  |  |  |  |  | *** |  |  |  |  | ns |
| None | 88.1 | 84.8 | 88.0 | 89.2 | 89.1 |  | 87.7 | 88.7 | 86.6 | 88.1 |  | 82.0 | 79.7 | 84.0 | 84.6 | 82.6 |  | 81.5 | 81.1 | 82.5 | 80.9 |  |
| Co-resident | 3.8 | 5.6 | 4.1 | 3.0 | 2.8 |  | 3.6 | 3.5 | 3.5 | 3.9 |  | 3.4 | 4.0 | 3.1 | 2.5 | 3.7 |  | 3.3 | 3.1 | 3.3 | 4.0 |  |
| Non-resident | 7.7 | 8.8 | 7.5 | 7.6 | 7.6 |  | 8.3 | 7.5 | 9.3 | 7.7 |  | 13.8 | 15.4 | 12.0 | 12.4 | 12.7 |  | 14.5 | 15.1 | 13.7 | 10.1 |  |
| At both locations | 0.4 | 0.9 | 0.4 | 0.2 | 0.5 |  | 0.4 | 0.3 | 0.6 | 0.3 |  | 0.8 | 0.9 | 0.8 | 0.5 | 1.0 |  | 6.9 | 0.7 | 0.5 | 1.0 |  |
| **Education attainment:** |  |  |  |  |  | *** |  |  |  |  | *** |  |  |  |  |  | *** |  |  |  |  | *** |
| Degree (or higher) | 43.2 | 39.0 | 42.7 | 45.6 | 43.1 |  | 42.6 | 47.3 | 44.7 | 32.3 |  | 48.7 | 40.4 | 50.4 | 62.7 | 71.9 |  | 47.3 | 46.4 | 55.2 | 37.1 |  |
| A level (or equivalent) | 25.0 | 24.7 | 26.0 | 24.5 | 24.2 |  | 24.5 | 21.7 | 24.7 | 28.2 |  | 20.8 | 21.4 | 22.0 | 19.2 | 10.7 |  | 20.4 | 21.0 | 17.2 | 24.0 |  |
| GCSE (or equivalent) | 20.2 | 20.4 | 20.2 | 20.0 | 20.1 |  | 20.4 | 18.7 | 18.9 | 25.5 |  | 20.6 | 25.0 | 19.7 | 12.5 | 11.6 |  | 21.4 | 21.2 | 18.7 | 26.1 |  |
| Other qualification | 7.6 | 9.2 | 7.4 | 6.9 | 8.5 |  | 8.0 | 7.9 | 7.9 | 8.4 |  | 6.3 | 8.1 | 5.2 | 3.8 | 4.8 |  | 6.8 | 7.0 | 5.8 | 8.0 |  |
| No qualification | 4.0 | 6.8 | 3.7 | 3.0 | 4.1 |  | 4.4 | 4.4 | 3.8 | 5.6 |  | 3.6 | 5.1 | 2.7 | 1.8 | 1.0 |  | 4.1 | 4.3 | 3.1 | 4.8 |  |
| **NS-SEC occupations:** |  |  |  |  |  | *** |  |  |  |  | *** |  |  |  |  |  | *** |  |  |  |  | *** |
| Manager/professional | 43.7 | 29.0 | 44.0 | 49.1 | 45.2 |  | 43.2 | 51.2 | 44.8 | 28.7 |  | 41.9 | 29.1 | 47.5 | 62.5 | 63.8 |  | 41.4 | 42.3 | 49.4 | 26.7 |  |
| Intermediate | 21.5 | 28.8 | 23.0 | 16.0 | 24.0 |  | 21.7 | 18.8 | 22.4 | 24.6 |  | 25.6 | 26.5 | 29.4 | 18.4 | 19.7 |  | 25.8 | 29.8 | 21.6 | 22.3 |  |
| Routine | 34.8 | 42.2 | 33.0 | 34.8 | 30.8 |  | 35.1 | 30.0 | 32.8 | 46.7 |  | 32.5 | 44.5 | 23.1 | 19.2 | 16.5 |  | 32.8 | 27.9 | 29.0 | 51.0 |  |
| **Equivalised household income:** |  |  |  |  |  | *** |  |  |  |  | *** |  |  |  |  |  | *** |  |  |  |  | *** |
| Quintile 5 (highest) | 22.5 | 14.9 | 18.1 | 27.3 | 31.5 |  | 22.3 | 23.4 | 25.4 | 15.4 |  | 20.8 | 13.8 | 21.8 | 33.8 | 39.5 |  | 20.7 | 20.3 | 26.0 | 13.4 |  |
| Quintile 4 | 21.0 | 14.9 | 21.4 | 22.9 | 21.7 |  | 21.0 | 23.9 | 21.1 | 16.5 |  | 20.9 | 16.3 | 24.7 | 26.5 | 22.8 |  | 20.6 | 22.1 | 20.0 | 17.8 |  |
| Quintile 3 | 20.7 | 17.1 | 22.9 | 20.6 | 18.5 |  | 20.4 | 21.2 | 19.3 | 20.9 |  | 20.5 | 19.4 | 24.2 | 18.6 | 16.2 |  | 20.6 | 21.0 | 20.4 | 19.7 |  |
| Quintile 2 | 20.0 | 21.2 | 22.3 | 18.2 | 17.0 |  | 20.4 | 18.0 | 20.3 | 24.3 |  | 20.7 | 24.9 | 18.9 | 14.3 | 12.2 |  | 20.9 | 21.4 | 18.5 | 23.5 |  |
| Quintile 1 (lowest) | 15.8 | 31.9 | 15.4 | 11.0 | 11.3 |  | 15.9 | 13.5 | 13.9 | 22.9 |  | 17.0 | 25.6 | 10.3 | 6.8 | 9.2 |  | 17.2 | 15.2 | 15.1 | 25.6 |  |
| **LLTI:** |  |  |  |  |  | *** |  |  |  |  | ns |  |  |  |  |  | * |  |  |  |  | ns |
| No | 76.9 | 69.6 | 77.8 | 78.7 | 77.6 |  | 76.8 | 75.3 | 78.3 | 76.4 |  | 74.3 | 73.1 | 75.5 | 75.2 | 77.1 |  | 74.2 | 74.4 | 74.1 | 73.7 |  |
| Yes | 23.1 | 30.4 | 22.2 | 21.2 | 22.4 |  | 23.2 | 24.7 | 21.7 | 23.6 |  | 25.7 | 26.9 | 24.5 | 24.8 | 22.9 |  | 25.8 | 25.6 | 25.9 | 26.3 |  |
| **Smoker status:** |  |  |  |  |  | *** |  |  |  |  | *** |  |  |  |  |  | ns |  |  |  |  | *** |
| Non-smoker | 42.4 | 39.2 | 44.7 | 42.4 | 38.8 |  | 40.7 | 42.4 | 40.4 | 38.7 |  | 49.0 | 48.7 | 50.0 | 47.8 | 52.4 |  | 47.1 | 50.2 | 45.6 | 41.8 |  |
| Ex-smoker | 37.1 | 40.0 | 35.9 | 37.3 | 36.8 |  | 37.2 | 37.9 | 37.9 | 34.8 |  | 32.8 | 33.5 | 31.5 | 33.1 | 31.1 |  | 33.1 | 33.4 | 33.9 | 31.3 |  |
| Smoker | 20.5 | 20.7 | 19.4 | 20.3 | 24.4 |  | 22.1 | 19.7 | 21.7 | 26.5 |  | 18.2 | 17.8 | 18.5 | 19.1 | 16.5 |  | 19.7 | 16.4 | 20.5 | 26.9 |  |
| **Exercise frequency:** |  |  |  |  |  | *** |  |  |  |  | * |  |  |  |  |  | *** |  |  |  |  | ns |
| >3 times/wk | 21.0 | 20.1 | 20.6 | 22.1 | 19.8 |  | 19.9 | 21.0 | 19.7 | 18.6 |  | 15.2 | 13.1 | 16.2 | 18.5 | 18.8 |  | 14.4 | 14.4 | 14.9 | 13.7 |  |
| 1-3 times/wk | 23.9 | 22.6 | 23.2 | 25.3 | 23.1 |  | 23.8 | 24.2 | 24.3 | 22.4 |  | 21.8 | 21.5 | 21.3 | 22.9 | 25.3 |  | 21.9 | 22.1 | 22.4 | 20.3 |  |
| <1 time/wk | 34.6 | 32.3 | 34.5 | 35.0 | 37.3 |  | 36.8 | 36.1 | 37.6 | 36.4 |  | 36.2 | 36.0 | 36.6 | 36.1 | 35.2 |  | 37.2 | 36.9 | 37.9 | 37.1 |  |
| No exercise | 20.5 | 25.0 | 21.7 | 17.6 | 19.8 |  | 19.5 | 18.7 | 18.4 | 22.6 |  | 26.8 | 29.4 | 25.9 | 22.5 | 20.7 |  | 26.5 | 26.6 | 24.8 | 28.9 |  |
| **Alcohol consumption frequency:** |  |  |  |  |  | *** |  |  |  |  | *** |  |  |  |  |  | *** |  |  |  |  | *** |
| None | 27.8 | 33.6 | 28.9 | 25.0 | 25.5 |  | 24.8 | 23.5 | 22.5 | 30.5 |  | 37.6 | 39.8 | 36.5 | 34.2 | 35.5 |  | 34.0 | 33.3 | 31.9 | 39.1 |  |
| 1-2 days/wk | 35.3 | 31.1 | 35.3 | 36.3 | 37.5 |  | 36.7 | 37.6 | 34.8 | 38.6 |  | 36.0 | 34.5 | 38.4 | 36.2 | 35.6 |  | 37.9 | 38.3 | 36.7 | 38.8 |  |
| 3-4 days/wk | 21.8 | 18.5 | 22.5 | 22.9 | 20.4 |  | 22.1 | 23.1 | 24.4 | 16.7 |  | 17.1 | 16.6 | 16.4 | 19.4 | 17.6 |  | 17.7 | 18.2 | 19.5 | 13.8 |  |
| ≥5 days/wk | 15.1 | 16.9 | 13.3 | 15.8 | 16.6 |  | 16.4 | 15.8 | 18.3 | 14.1 |  | 9.3 | 9.1 | 8.6 | 10.2 | 11.4 |  | 10.3 | 10.2 | 11.9 | 8.3 |  |
| **Job satisfaction:** |  |  |  |  |  | *** |  |  |  |  | * |  |  |  |  |  | ns |  |  |  |  | ns |
| Satisfied | 78.1 | 78.6 | 76.0 | 78.8 | 81.7 |  | 76.8 | 75.1 | 78.4 | 76.4 |  | 80.0 | 80.8 | 78.9 | 79.6 | 80.5 |  | 78.5 | 78.7 | 78.9 | 77.4 |  |
| Neutral | 9.6 | 9.2 | 10.3 | 9.6 | 7.7 |  | 10.1 | 10.6 | 9.7 | 10.0 |  | 8.0 | 8.0 | 8.2 | 7.6 | 7.3 |  | 8.4 | 8.6 | 7.6 | 9.3 |  |
| Dissatisfied | 12.3 | 12.2 | 13.7 | 11.6 | 10.6 |  | 13.1 | 14.3 | 11.9 | 13.6 |  | 12.0 | 11.1 | 12.8 | 12.8 | 12.3 |  | 13.1 | 12.7 | 13.5 | 13.3 |  |
| **Income satisfaction:** |  |  |  |  |  | *** |  |  |  |  | *** |  |  |  |  |  | *** |  |  |  |  | *** |
| Satisfied | 60.1 | 55.9 | 58.9 | 62.6 | 61.8 |  | 54.6 | 57.5 | 55.5 | 48.9 |  | 58.1 | 55.4 | 59.7 | 61.6 | 63.6 |  | 52.1 | 53.8 | 53.7 | 45.5 |  |
| Neutral | 12.4 | 13.6 | 12.8 | 11.9 | 11.0 |  | 12.8 | 11.4 | 12.8 | 14.6 |  | 11.9 | 13.2 | 10.8 | 10.6 | 10.6 |  | 12.4 | 11.7 | 11.3 | 15.7 |  |
| Dissatisfied | 27.5 | 30.5 | 28.3 | 25.5 | 27.2 |  | 32.6 | 31.1 | 31.7 | 36.5 |  | 30.0 | 31.4 | 29.5 | 27.8 | 25.8 |  | 35.5 | 34.5 | 35.0 | 38.8 |  |
| **Leisure satisfaction:** |  |  |  |  |  | *** |  |  |  |  | *** |  |  |  |  |  | *** |  |  |  |  | *** |
| Satisfied | 54.4 | 66.7 | 57.1 | 50.9 | 39.9 |  | 52.0 | 57.3 | 51.8 | 44.6 |  | 54.3 | 58.5 | 55.4 | 45.6 | 34.7 |  | 51.0 | 54.0 | 51.0 | 43.6 |  |
| Neutral | 14.5 | 13.8 | 15.2 | 14.6 | 13.0 |  | 14.6 | 14.8 | 13.9 | 15.6 |  | 13.5 | 14.6 | 12.9 | 12.2 | 10.9 |  | 14.2 | 14.0 | 12.8 | 17.0 |  |
| Dissatisfied | 31.1 | 19.5 | 27.8 | 34.5 | 47.1 |  | 34.4 | 27.9 | 34.3 | 39.8 |  | 32.2 | 26.9 | 31.7 | 42.3 | 54.4 |  | 34.8 | 32.0 | 36.2 | 39.4 |  |
| **Job physicality:** |  |  |  |  |  | *** |  |  |  |  | *** |  |  |  |  |  | *** |  |  |  |  | *** |
| Not at all | 24.7 | 27.3 | 22.7 | 24.9 | 27.2 |  | 24.9 | 18.0 | 24.9 | 35.0 |  | 20.2 | 21.9 | 17.6 | 18.2 | 28.1 |  | 20.4 | 15.2 | 20.1 | 33.7 |  |
| Not very | 36.8 | 38.4 | 35.4 | 36.7 | 39.0 |  | 37.0 | 33.0 | 37.6 | 41.7 |  | 39.5 | 42.2 | 35.6 | 38.6 | 40.5 |  | 39.7 | 36.4 | 40.9 | 46.0 |  |
| Fairly | 24.5 | 22.7 | 25.0 | 25.1 | 23.8 |  | 24.7 | 29.9 | 25.3 | 16.1 |  | 26.0 | 23.2 | 26.4 | 29.0 | 21.5 |  | 26.1 | 30.9 | 26.0 | 14.4 |  |
| Very | 14.0 | 11.6 | 16.9 | 13.3 | 10.0 |  | 13.4 | 19.1 | 12.2 | 7.2 |  | 14.3 | 12.7 | 17.4 | 14.3 | 9.9 |  | 13.8 | 17.5 | 13.0 | 5.9 |  |
| **Work autonomy:** |  |  |  |  |  | *** |  |  |  |  | *** |  |  |  |  |  | *** |  |  |  |  | *** |
| Mean (SD) | 11.8 (4.0) | 11.2  (4.5) | 11.6  (3.9) | 12.0  (3.8) | 12.6  (3.8) |  | 11.7  (4.0) | 11.7  (3.9) | 11.9  (3.9) | 11.4  (4.3) |  | 11.0 (4.0) | 10.7  (4.2) | 11.2  (3.8) | 11.6  (3.7) | 11.8  (3.9) |  | 10.9  (4.1) | 10.9  (3.9) | 11.2  (4.1) | 10.6  (4.3) |  |

^a^ by percent unless otherwise stated. ^b^ uses data from w4 & w7 pooled samples. ^c^ uses data from w4-only sample. Only temporal work patterns which had an interaction with gender are shown. Data are multiply-imputed separately be gender. Sample sizes are unweighted. Survey weights were applied in all analyses. ***p<0.001, **p<0.01, *p<0.05 significant differences are between categories of each work pattern.
