# Supplementary material for: Work hours, weekend working, nonstandard work schedules and sleep quantity and quality: findings from the UK household longitudinal study
Source: BMC Public Health. 2024 Jan 27;24:309. doi: 10.1186/s12889-024-17762-0 (PMC10821573; doi:10.1186/s12889-024-17762-0)
Supplement: Supplementary file 3 — Additional file 3: Table A3. Associations between combinations of temporal work patterns and sleep durationa, and sleep disturbanceb. This is a table showing the regression results for the associations between different combinations of temporal work patterns and sleep. [file 12889_2024_17762_MOESM3_ESM.docx]

Additional file 3.docx

Table A3 Associations between combinations of temporal work patterns and sleep duration^a^, and sleep disturbance^b^

|  | **Participants** |  | **Sleep duration (ref: 7-8 hrs/night)** | | | | | | | | **Sleep disturbance (ref: no disturbance)** | | | |
| --- | --- | --- | --- | --- | --- | --- | --- | --- | --- | --- | --- | --- | --- | --- |
|  | **N=25605^c^** |  | **Model 1** | | | | **Model 2** | | | | **Model 1** | | **Model 2** | |
|  |  |  | **<7 hrs/night** | | **≥9 hrs/night** | | **<7 hrs/night** | | **≥9 hrs/night** | | **Sleep disturbance** | | **Sleep disturbance** | |
|  | **%** |  | **OR** | **95% CI** | **OR** | **95% CI** | **OR** | **95% CI** | **OR** | **95% CI** | **OR** | **95% CI** | **OR** | **95% CI** |
| **Work pattern (ref: 35-40 hours/week & standard schedules & non-weekends)** | 17.2 |  | 1.00 |  | 1.00 |  | 1.00 |  | 1.00 |  | 1.00 |  | 1.00 |  |
| **Panel A: One atypical temporal work pattern**: |  |  |  |  |  |  |  |  |  |  |  |  |  |  |
| <35 hours/week & standard schedules & non-weekends | 13.8 |  | 0.94 | 0.83, 1.06 | 1.22 | 0.84, 1.76 | 0.98 | 0.86, 1.11 | 1.20 | 0.83, 1.75 | 1.06 | 0.92, 1.22 | 1.13 | 0.98, 1.31 |
| 41-54 hours/week & standard schedules & non-weekends | 6.9 |  | 1.23 | 1.06, 1.42 | 0.91 | 0.56, 1.49 | 1.23 | 1.06, 1.42 | 0.91 | 0.56, 1.48 | 1.09 | 0.92, 1.29 | 1.08 | 0.91, 1.29 |
| ≥55 hours/week & standard schedules & non-weekends | 0.7 |  | 1.70 | 1.13, 2.56 | 0.16 | 0.02, 1.21 | 1.65 | 1.09, 2.47 | 0.15 | 0.02, 1.14 | 1.37 | 0.88, 2.12 | 1.26 | 0.81, 1.97 |
| 35-40 hours/week & standard schedules & some weekends | 8.3 |  | 1.14 | 0.98, 1.33 | 0.82 | 0.47, 1.41 | 1.16 | 1.00, 1.35 | 0.80 | 0.46, 1.39 | 1.10 | 0.92, 1.32 | 1.11 | 0.93, 1.33 |
| 35-40 hours/week & standard schedules & most/all weekends | 2.3 |  | 1.30 | 1.03, 1.66 | 1.52 | 0.76, 3.05 | 1.31 | 1.03, 1.68 | 1.49 | 0.74, 2.99 | 1.17 | 0.89, 1.54 | 1.16 | 0.88, 1.53 |
| 35-40 hours/week & nonstandard & non-weekends | 1.1 |  | 1.45 | 1.02, 2.07 | 1.70 | 0.73, 3.95 | 1.44 | 1.01, 2.05 | 1.68 | 0.72, 3.91 | 1.36 | 0.93, 1.98 | 1.35 | 0.92, 1.98 |
|  |  |  |  |  |  |  |  |  |  |  |  |  |  |  |
| **Panel B: Two atypical temporal work patterns:** |  |  |  |  |  |  |  |  |  |  |  |  |  |  |
| <35 hours/week & standard schedules & some weekends | 5.3 |  | 1.06 | 0.90, 1.25 | 1.39 | 0.85, 2.26 | 1.11 | 0.94, 1.32 | 1.38 | 0.84, 2.24 | 1.16 | 0.96, 1.39 | 1.22 | 1.01, 1.47 |
| 41-54 hours/week & standard schedules & some weekends | 8.5 |  | 1.23 | 1.06, 1.41 | 1.04 | 0.63, 1.71 | 1.21 | 1.05, 1.39 | 1.01 | 0.61, 1.67 | 1.19 | 1.01, 1.41 | 1.16 | 0.98, 1.37 |
| ≥55 hours/week & standard schedules & some weekends | 1.6 |  | 2.00 | 1.52, 2.63 | 0.77 | 0.26, 2.22 | 1.90 | 1.44, 2.51 | 0.75 | 0.26, 2.17 | 1.28 | 0.93, 1.76 | 1.16 | 0.85, 1.60 |
| <35 hours/week & standard schedules & most/all weekends | 3.0 |  | 1.34 | 1.08, 1.66 | 2.14 | 1.32, 3.48 | 1.38 | 1.11, 1.71 | 2.14 | 1.31, 3.47 | 1.09 | 0.86, 1.36 | 1.11 | 0.88, 1.40 |
| 41-54 hours/week & standard schedules & most/all weekends | 2.8 |  | 1.21 | 0.97, 1.51 | 1.44 | 0.80, 2.61 | 1.16 | 0.93, 1.44 | 1.37 | 0.75, 2.51 | 1.12 | 0.87, 1.43 | 1.03 | 0.81, 1.32 |
| ≥55 hours/week & standard schedules & most/all weekends | 1.3 |  | 1.71 | 1.26, 2.32 | 1.23 | 0.47, 3.23 | 1.62 | 1.19, 2.19 | 1.15 | 0.44, 3.01 | 1.06 | 0.74, 1.53 | 0.95 | 0.66, 1.37 |
| <35 hours/week & nonstandard & non-weekends | 1.7 |  | 1.00 | 0.77, 1.32 | 1.33 | 0.70, 2.54 | 1.07 | 0.81, 1.40 | 1.32 | 0.69, 2.52 | 0.98 | 0.72, 1.32 | 1.06 | 0.78, 1.45 |
| 41-54 hours/week & nonstandard & non-weekends | 0.7 |  | 2.43 | 1.62, 3.64 | 1.93 | 0.75, 4.97 | 2.38 | 1.58, 3.58 | 1.85 | 0.71, 4.83 | 1.56 | 1.02, 2.38 | 1.50 | 0.97, 2.33 |
| ≥55 hours/week & nonstandard & non-weekends | 0.2 |  | 2.88 | 1.29, 6.43 | 2.58 | 0.31, 21.6 | 2.63 | 1.19, 5.81 | 2.59 | 0.31, 21.6 | 2.23 | 1.08, 4.60 | 2.06 | 1.01, 4.21 |
| 35-40 hours/week & nonstandard & some weekends | 3.5 |  | 1.41 | 1.14, 1.73 | 1.45 | 0.81, 2.58 | 1.40 | 1.13, 1.72 | 1.39 | 0.78, 2.47 | 1.26 | 0.99, 1.60 | 1.26 | 0.99, 1.60 |
| 35-40 hours/week & nonstandard & most/all weekends | 2.8 |  | 1.62 | 1.30, 2.02 | 1.75 | 1.05, 2.93 | 1.56 | 1.25, 1.95 | 1.71 | 1.02, 2.88 | 1.69 | 1.34, 2.13 | 1.62 | 1.27, 2.05 |
|  |  |  |  |  |  |  |  |  |  |  |  |  |  |  |
| **Panel C: Three atypical work patterns:** |  |  |  |  |  |  |  |  |  |  |  |  |  |  |
| <35 hours/week & nonstandard & some weekends | 3.8 |  | 1.19 | 0.99, 1.44 | 1.51 | 0.93, 2.47 | 1.24 | 1.02, 1.50 | 1.48 | 0.90, 2.42 | 1.14 | 0.93, 1.40 | 1.19 | 0.97, 1.47 |
| 41-54hours/week & nonstandard & some weekends | 3.8 |  | 1.64 | 1.37, 1.98 | 0.75 | 0.38, 1.48 | 1.63 | 1.35, 1.96 | 0.72 | 0.36, 1.41 | 1.52 | 1.23, 1.88 | 1.51 | 1.22, 1.87 |
| ≥55 hours/week & nonstandard & some weekends | 1.6 |  | 1.68 | 1.25, 2.25 | 1.35 | 0.57, 3.19 | 1.61 | 1.20, 2.16 | 1.28 | 0.54, 3.03 | 1.42 | 1.03, 1.95 | 1.35 | 0.98, 1.86 |
| <35 hours/week & nonstandard & most/all weekends | 3.6 |  | 1.20 | 0.98, 1.48 | 2.18 | 1.44, 3.29 | 1.20 | 0.98, 1.48 | 2.10 | 1.39, 3.18 | 1.44 | 1.16, 1.80 | 1.46 | 1.16, 1.82 |
| 41-54 hours/week & nonstandard & most/all weekends | 3.0 |  | 1.56 | 1.26, 1.94 | 0.97 | 0.48, 1.94 | 1.50 | 1.21, 1.86 | 0.93 | 0.46, 1.87 | 1.31 | 1.03, 1.68 | 1.23 | 0.95, 1.58 |
| ≥55 hours/week & nonstandard & most/all weekends | 2.5 |  | 1.97 | 1.55, 2.50 | 1.27 | 0.61, 2.63 | 1.86 | 1.46, 2.38 | 1.18 | 0.56, 2.49 | 1.37 | 1.06, 1.78 | 1.24 | 0.95, 1.62 |

^a^ multinomial logistic regression analyses. ^b^ logistic regression analyses. ^c^ w4-only sample. Data are multiply imputed. Sample sizes are unweighted. Survey weights were applied to percentages and in regression analyses.

Model 1 adjusted for gender, age, age-squared, marital status, youngest child in the household, caregiving, housing tenure, educational attainment, equivalised household income, NS-SEC, smoker status, exercise frequency, and frequency of alcohol consumption.

Model 2 = model 1 + work conditions: job satisfaction, satisfaction with income, satisfaction with leisure time, work autonomy, and job physicality.
